# Emotional Reactions Mediate the Effect of Music Listening on Creative Thinking: Perspective of the Arousal-and-Mood Hypothesis

**DOI:** 10.3389/fpsyg.2017.01680

**Published:** 2017-09-26

**Authors:** Wu-Jing He, Wan-Chi Wong, Anna N.-N. Hui

**Affiliations:** ^1^Department of Special Education and Counselling, Education University of Hong Kong, Hong Kong, Hong Kong; ^2^Department of Educational Psychology, Chinese University of Hong Kong, Hong Kong, Hong Kong; ^3^Department of Applied Social Sciences, City University of Hong Kong, Hong Kong, Hong Kong

**Keywords:** emotional reactions, music listening, creative thinking, arousal-and-mood hypothesis, mediating effect

## Abstract

This study examined the effect of music listening on creative thinking through the lens of the arousal-and-mood hypothesis, which posits that emotional reactions (i.e., arousal and valence) mediate the effect of music listening on cognitive functioning. Participants were randomly assigned to three groups: a positive music group (*n* = 198), a negative music group (*n* = 195), and a control group (*n* = 191). Creative thinking and emotional reactions were assessed with the Test for Creative Thinking-Drawing Production and the Affect Grid, respectively. The results showed that both positively and negatively arousing music enhanced creative thinking. The results further revealed that arousal, regardless of valence, significantly mediated the music-creativity relationship. This study enriches the research on the arousal-and-mood hypothesis by (1) providing direct empirical testing on the mediating roles of emotional reactions; (2) including both positively and negatively arousing music in the study design; and (3) identifying that only arousal, and not valence, was a significant mediator in the music-creativity link.

## Introduction

The role of music in cognitive functioning is one of the most popular issues of discussion in research (Corrigall et al., 2013). Widespread interest in the potential benefits of listening to music was sparked by a series of studies regarding the *Mozart effect* (Jones and Estell, 2007), which documented that listening to music composed by Mozart led to significant improvements in spatial intelligence (e.g., Rauscher et al., 1993, 1995). The Mozart effect captured widespread attention in education, public policy, and the community even though subsequent empirical studies regarding the Mozart effect yielded inconsistent findings (Pietschnig et al., 2010). Researchers have been increasingly interested in the possible impacts of music exposure on cognitive functioning, including intelligence and creativity (Schellenberg, 2005; Schellenberg et al., 2007; Swaminathan et al., 2017). Joining this line of research, the present study aimed to examine the effect of music listening on creativity through the lens of the *arousal-and-mood hypothesis*.

### The Arousal-and-Mood Hypothesis

The arousal-and-mood hypothesis was initially proposed to account for the mixed findings regarding the Mozart effect (e.g., Thompson et al., 2001; Husain et al., 2002; Schellenberg et al., 2007). The hypothesis postulates that music listening does not function on the subsequent cognitive functioning directly. Rather, music listening affects subsequent cognitive functioning through the *indirect* effect (or the *mediation* effect) of emotional reactions. In other words, listening to music does not necessarily lead to an improvement in subsequent cognitive functioning; rather, individuals’ emotional reactions play a critical role that determines whether music listening has a beneficial effect on subsequent cognitive functioning.

In the arousal-and-mood hypothesis, emotional reactions were defined according to two orthogonal dimensions in the circumplex model of emotions (Russell, 1980; Russell et al., 1989; Russell and Carroll, 1999), with one dimension corresponding to *arousal* and the other to *mood*. As Husain et al. (2002) put it, “Arousal and mood represent different but related aspects of emotional responding… Arousal and mood correspond closely to *activation* and *valence*, respectively, which are the two orthogonal dimensions in Russell’s (1980) *circumplex model* of emotions” (p. 153). Specifically, arousal was defined as the degree of physical and psychological activation, i.e., the intensity of the felt emotion, and mood was defined as the valence of the felt emotion, i.e., positive or negative (Husain et al., 2002; Schellenberg et al., 2007). This conceptualization of emotional reactions is in line with most literature on mood, affect, and emotion, in which an emotion is also parsed into two dimensions: arousal/activation and valence/hedonic tone (e.g., Baas et al., 2008; De Dreu et al., 2008; Gilet and Jallais, 2011).

In explaining the underlying mechanism regarding the effect of music exposure on subsequent cognitive functioning, the arousal-and-mood hypothesis posits that “listening to music affects arousal and mood, which then influence performance on various cognitive skills” (Husain et al., 2002, p. 153). In a similar vein, Schellenberg (2005) made it clear that music listening is not the determining factor that functions on the subsequent cognitive functioning; rather, it (music listening) represents only “one example of a stimulus that influences the perceiver’s arousal level and mood, which can affect performance on a variety of cognitive tasks” (p. 318). Schellenberg (2005) further elaborated, “…music listening can lead to enhanced performance on a variety of tests of cognitive ability. These effects are mediated by arousal and mood and are unlikely to differ from those that arise as a consequence of exposure to non-musical stimuli that have similar emotional impact…” (p. 318).

Furthermore, the arousal-and-mood hypothesis seems to put a specific emphasis on the importance of a *moderate arousal level* and a *positive valence* of emotional reactions in subsequent cognitive functioning. As Husain et al. (2002) put it, “… virtually any moderately arousing stimulus that induces positive moods should affect performance on a variety of cognitive tasks, similar to the effect on spatial abilities that occurs as a consequence of listening to music composed by Mozart” (p. 167). Following this logic, it has been argued that any pleasant or enjoyable stimulus, either musical or non-musical, that arouses a positive hedonic tone at a moderate level can enhance the performance of cognitive functioning (Schellenberg et al., 2007). Indeed, some indirect empirical evidence are available to support the arousal-and-mood hypothesis (primarily in the intelligence domain). Such research findings generally illustrated that as long as the music was positively arousing, a parallel improvement was also evident in the subsequent performance on an intelligence test (e.g., Chabris, 1999; Nantais and Schellenberg, 1999; Steele et al., 1999; Steele, 2000; Thompson et al., 2001; Husain et al., 2002; Schellenberg, 2005; Schellenberg et al., 2007).

The study of the arousal-and-mood hypothesis is important because it offers an explanation as to why some individuals benefit from music listening and enhance their cognitive performance after music exposure, whereas others do not. The arousal-and-mood hypothesis highlights that it is an individual’s emotional reaction to the experience of music listening that determines the beneficial effect. This explanation helps explain the inconsistent findings regarding the Mozart effect. It also provides a framework to understand for whom and under what circumstances music listening enhances cognitive functioning and the mechanisms through which music exposure is effective.

### Extending the Study of the Arousal-and-Mood Hypothesis to Creativity

The study of the arousal-and-mood hypothesis is important, and previous empirical examinations of it have predominantly focused on intelligence. Thus, the present study aimed to extend the research on this hypothesis to creativity. Creativity, commonly conceptualized as consisting of originality and appropriateness, has been regarded as a key human resource for both personal and societal success (Sternberg and Lubart, 1999; Sternberg et al., 2005). Empirical research findings suggest that creative thinking is correlated with intelligence to some degree (e.g., Nusbaum and Silvia, 2011; Jauk et al., 2013; Karwowski et al., 2016). These conceptualizations and research findings suggest that creativity and intelligence are two different psychological constructs that are interrelated to a certain extent. Because the arousal-and-mood hypothesis posits that the effect of music exposure should be replicable with other tests of cognitive functioning, it is expected that the hypothesis can also be generalized to the domain of creativity. As one of the authors of the arousal-and-mood hypothesis, Schellenberg (2005) argued, “Listening to music composed by Mozart does not have unique or special consequences for spatial abilities. Rather, upbeat, age-appropriate music can improve listeners’ arousal level and mood… In turn, effects of arousal and mood extend beyond measures of spatial ability to tests of processing speed and creativity” (p. 318).

Given that the existing empirical evidence for the arousal-and-mood hypothesis is predominantly in the intelligence domain, extending the study of the hypothesis to the creativity domain would help examine the generalizability of the hypothesis. The existing literature seems to lack direct empirical examinations of how emotional reactions mediate the effect of music exposure on creativity. However, three different lines of relevant research work appear to support the possibility of the mediation effect. The first line of research concerns the effect of music exposure and creativity. Studies demonstrate a positive relationship between music exposure and enhanced performance on creativity measures (e.g., Kavanagh, 1987; Adaman and Blaney, 1995; Moga et al., 2000). The second line of research documents a link between music exposure and emotion induction. These studies consistently demonstrate that music exposure is effective in inducing emotions (e.g., Schellenberg et al., 2007; Zenasni and Lubart, 2008). Finally, the third line of research is based on the rich literature on the mood-creativity link. A large body of research suggests that aroused emotions significantly enhance creative functioning (e.g., Ashby et al., 1999; Baas et al., 2008, 2011; De Dreu et al., 2008; Hirt et al., 2008). In summary, an integration of these three separate lines of evidence suggests the possible mediation effect of emotional reactions on the music-creativity link.

Indeed, Schellenberg et al. (2007) reported initial (though indirect) empirical evidence to support the arousal-and-mood hypothesis in the creativity domain. They showed that 5-year-old Japanese children obtained higher scores on a creative drawing task subsequent to listening to familiar songs or singing songs that they liked. The research finding of Schellenberg et al. (2007) was regarded as indirect empirical evidence because the authors did not include a direct measure of children’s emotional reactions in response to their music experience. Due to the lack of a direct measure of children’s emotional reactions, no relevant data were available for conducting statistical analyses regarding a mediation effect. It remains unclear whether the children’s performance on the drawing task was enhanced through the mediation effect of the positively aroused emotions or not. Hence, the present study aimed to advance the design of Schellenberg et al.’s (2007) study by employing a standardized measure of emotional reactions, the Affect Grid, to assess changes in arousal and valence with respect to the music listening experience (see section “The Affect Grid”). By obtaining data on the changes in arousal or valence in response to music listening, the mediation effect of arousal and valence on the relationship between music and creativity can be directly tested.

### Extending the Study of the Arousal-and-Mood Hypothesis Using Both Positively and Negatively Arousing Music

The second aim of this study was to examine the arousal-and-mood hypothesis in the creativity domain using both positively and negatively arousing music. As discussed in Section “The Arousal-and-Mood Hypothesis,” the arousal-and-mood hypothesis puts a specific emphasis on the critical role of two emotional reaction factors, (1) moderate arousal level and (2) positive valence, in the contribution to the enhancement of cognitive functioning subsequent to listening to music (Husain et al., 2002; Schellenberg, 2005; Schellenberg et al., 2007). However, this theoretical notion appears to be inconsistent with the existing literature with respect to the mood-creativity relationship. Although many researchers, in line with the arousal-and-mood hypothesis, have highlighted the facilitative roles of positive emotions in creativity (e.g., Isen et al., 1987; Fredrickson, 1998; Ashby et al., 1999; Lyubomirsky et al., 2005; Isen, 2008), other researchers have argued that both positively and negatively valenced emotional reactions contribute to creativity (e.g., Schwarz, 1990; Baas et al., 2008, 2011; De Dreu et al., 2008; Davis, 2009; Forgeard, 2011).

For example, in their dual pathway to creativity model, Baas et al. (2008) postulate that both positive and negative hedonic tones contribute to creativity through different routes. They suggest that “activating moods that are positive in tone increase creative fluency and originality primarily through enhanced cognitive flexibility, whereas activating moods that are negative in tone increase creative fluency and originality primarily through enhanced persistence and perseverance” (Baas et al., 2008; p. 742). In a similar vein, Schwarz (1990), in their feelings-as-information theory, argued that both positive and negative emotions could contribute to creativity by eliciting different types of information-processing strategies. Positive emotions may indicate a state of well-being and are therefore accompanied by a relaxed and playful approach to information processing, which is favorable for idea generation. On the contrary, negative emotions may indicate the presence of danger and therefore require systematic, detail-oriented thinking strategies that may help with idea evaluation. Both idea generation and idea evaluation contribute significantly to creative thinking. Indeed, many empirical studies regarding the relationship between induced emotions and creativity suggest that not only positive emotion but also negative emotion can enhance creative thinking (see Baas et al., 2008, 2011; De Dreu et al., 2008; Forgeard, 2011; Jovanovic et al., 2016; for a detailed review).

The research findings regarding the complex relationship between mood and creativity led to a challenge to the notion that a positive emotional state is more important than a negative emotional state in the facilitation of creative performance (Kaufmann and Vosburg, 1997; George and Zhou, 2002; Gasper, 2003; Shalley et al., 2004). This challenge is also applicable to the prediction of the arousal-and-mood hypothesis with respect to the role of positive arousal valence in creative thinking. The question arises regarding whether the arousal-and-mood hypothesis is limited to positively aroused emotions. It is interesting to investigate whether the arousal-and-mood hypothesis can be generalized to negatively aroused emotions, as suggested by the literature on the mood-creativity relationship. Addressing this question can help enrich the discourse on the arousal-and-mood hypothesis and enhance the understanding of the effect of music listening on creative thinking.

In their empirical attempt to examine the arousal-and-mood hypothesis in the creativity domain, Schellenberg et al. (2007) merely illustrated the effect of a possible positively aroused emotion by asking their participants to listen to familiar songs or sing songs that they liked. It remains unclear whether similar enhancements in creativity can be observed if negatively arousing music is used. Hence, in the present study, we aimed to take a step further to examine the arousal-and-mood hypothesis in the domain of creativity using both positively and negatively arousing music.

### Hypotheses

Derived from the arousal-and-mood hypothesis and previous research findings on music, mood, and creativity, two hypotheses were generated: (1) music listening enhances creative thinking, and (2) emotional reactions (i.e., arousal and valence) mediate the effect of music listening on creative thinking.

## Materials and Methods

### Participants

A total of 584 schoolchildren (53.9% girls) in grades 4 through 9 and between the ages of 9 and 14 years (*M* = 11.2, *SD* = 0.96) were recruited on a voluntary basis from four co-educational schools in various districts of Hong Kong. All four schools admitted students from diverse backgrounds, but most students were from middle-class to lower-middle-class socioeconomic backgrounds. All participants were ethnic Chinese.

### Stimuli

The stimuli used in the present study consisted of 10-min excerpts of music that had been shown to successfully induce positive (i.e., Mozart’s Sonata for Two Pianos in D Major) or negative emotions (i.e., Albinoni’s Adagio in G Minor) in past studies (e.g., Chabris, 1999; Nantais and Schellenberg, 1999; Steele et al., 1999; Steele, 2000; Thompson et al., 2001; Husain et al., 2002; Schellenberg et al., 2007). The music excerpts were digitally recorded from compact disks onto the hard disk of a computer without a loss of sound quality.

### Instruments

#### Test for Creative Thinking-Drawing Production (TCT-DP)

The Test for Creative Thinking-Drawing Production (TCT-DP; Urban and Jellen, 1995/2010) was employed in this study to assess changes in creativity in response to music listening. The TCT-DP was developed based on a componential model of creativity (Jellen and Urban, 1986; Urban, 1991; Urban and Jellen, 1995/2010), which postulates that creativity involves the dynamic interaction among cognitive (e.g., divergent thinking, general and specific knowledge and skills) and personality components (e.g., task commitment, motivation, openness, and tolerance of ambiguity). In essence, creativity is assessed by performance in a drawing production with six intriguing figural fragments, including (a) a semicircle, (b) a point (c), a 90° angle, (d) a curved line, (e) a broken line, and (f) a small open square. The TCT-DP contains two parallel forms: Forms A and B. Both forms contain the same six test elements, where Form B is a 180° inversion of Form A. The drawing can be completed using any combination of the six figural fragments in a wide variety of ways, ranging from simple, conventional, and disjointed completions to thematically complex, unconventional, integrated, and aesthetically interesting completions (Dollinger et al., 2004). The instruction to complete the drawing with the given fragments was translated into Chinese with a back-translation procedure.

The criteria of the TCT-DP that we applied for assessing creativity in this study include the following: (1) *Continuations* involved any use or extension of the six fragments; (2) *Completions* involved any addition to the six continuations; (3) *New elements* referred to any new figures or symbols; (4) Connections that were made with a line (*Connections*[*Line*]) were scored based on the physical linkages between the continuations or completions of the given fragments and the new elements; (5) Connections that were made that produced a theme (*Connections*[*Theme*]) and involved any element or figure that contributed to a compositional theme; (6) *Boundary breaking* [*Fragment-dependent*] involved the use of a small open square that was located outside of the large square frame; (7) *Boundary breaking* [*Fragment-independent*] involved non-accidental drawing outside of the frame, excluding the use of the small open square; (8) *Perspective* was scored on the basis of the inclusion of three-dimensional elements; (9) *Humor and Affectivity* were scored on the basis of a drawing that expressed humor or other emotions; and (10) *Unconventionality* was scored according to four subcategories that included (a) manipulations of the materials, (b) surreal or abstract drawings, (c) atypical combinations of figures and symbols, and (d) non-stereotypical use of a certain element. The final criterion, *Speed*, was not applied because the test was administered in group mode. A composite score was obtained by summing the points for each of the aforementioned 10 criteria with no transformations. The possible score range was 0–6 points for each of the first nine criteria. Each of the four subcategories in the 10th criterion (Unconventionality) was scored according to a possible score range of 0–3 points. Thus, the total possible score range of the TCT-DP, excluding the criterion Speed, was 0–66 points; a higher score indicated higher levels of creativity.

The TCT-DP has been widely used with satisfactory validity and reliability for assessing creativity (Cropley, 2000; Dollinger et al., 2004; Lubart et al., 2010). The applicability of the instrument in Chinese samples has also been supported in previous studies (Rudowicz, 2004; He and Wong, 2011, 2015; He et al., 2013, 2017). In this study, reasonably good internal consistency statistics were obtained, with Cronbach’s alphas of 0.71 and 0.76 for Forms A and B, respectively. Moreover, an inter-rater reliability analysis was performed using Pearson’s correlation by two experienced raters who were blind to the study score 100 TCT-DP protocols. A high inter-rater correlation coefficient (*r* = 0.94; *p* < 0.001) was obtained for the composite score of the TCT-DP, which is comparable to the values reported in the testing manual (0.89 < *r* < 0.97, Urban and Jellen, 1995/2010).

#### The Affect Grid

The back-translated Affect Grid was employed to assess emotional reactions. The Affect Grid (Russell et al., 1989) was designed based on the theoretical basis of the circumplex model of emotions (Russell, 1980) to measure the emotional state along two dimensions: arousal-sleepiness (i.e., arousal) and pleasure-displeasure (i.e., mood). The Grid can be used rapidly and repeatedly to capture rapid fluctuations in the emotional states that emerge, for example, in response to music. Previous studies have also supported the validity of the Grid by showing significant correlations between the Affect Grid and similar measures of mood and affect, such as the Positive and Negative Affect Schedule and the Profile of Mood States (e.g., Killgore, 1998). Furthermore, the Affect Grid is considered a valid measure that can be used to assess mood and arousal in a quick and easy manner (Russell and Gobet, 2012).

**FIGURE 1:**
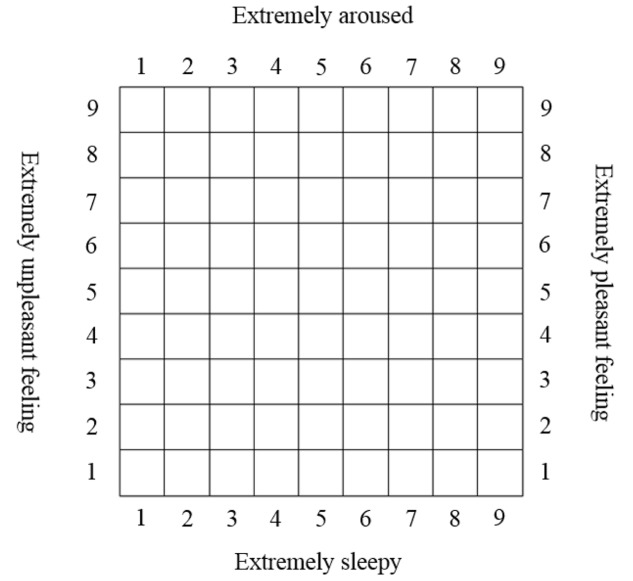
The Affect Grid (adapted from Russell et al., 1989).

**Figure 1** shows a sample Affect Grid. The center of the square represents a neutral, average, everyday feeling that is neither positive nor negative. The vertical dimension of the map represents the degree of arousal. The top half represents feelings that are above average in arousal, whereas the lower half represents feelings that are below average. Going from the bottom to the top represents a transition from a minimum arousal to a maximum arousal. Regarding the horizontal dimension of the map, the right half of the grid represents pleasant feelings (the farther to the right, the more pleasant), whereas the left half represents unpleasant feelings (the farther to the left, the more unpleasant). The participants were instructed to place a single mark within the grid to indicate their emotional state. The arousal score (A), which ranges from 1 to 9, is the number of the row checked, counting from the bottom. The pleasure score (P), which also ranges from 1 to 9, is the number of the column checked, counting from the left.

### Procedure

Two weeks prior to data collection, students were solicited for the study, which was described as measuring the relationship between music and thinking skills, and they were given a consent form to sign. The students were assured that all data collected would be strictly confidential and used for research purposes only. Only students who returned signed consent forms were invited to participate in the study.

At the beginning of the experiment, participants were administered the TCT-DP (Form A) and the Affect Grid as a pre-test to measure their baseline level of creativity and emotional state. In addition, participants were invited to complete a questionnaire that was designed to collect data on each participant’s (a) background information on music exposure (including previous musical education and music listening) and (b) demographic information (e.g., age, gender). Taking into account their performance on the creativity tests, the Affect Grid, and their music exposure or experience, the participants were assigned to three groups of the “equivalent creative potential and emotional states,” which included the positive music group (listening to music that arouses positive emotion; *n* = 198, 53.5% girls), the negative music group (listening to music that arouses negative emotion; *n* = 195, 54.4% girls), and the control group (sitting in silence; *n* = 191, 53.9% girls). The three groups were matched in terms of their creativity (*F*[2,581] = 0.12, *p* = 0.89), emotional state (Arousal: *F*[2,581] = 0.60, *p* = 0.55*;* Valence*: F*[2,581] = 1.01, *p* = 0.37), age (*F*[2,581] = 1.82, *p* = 0.16), gender distribution (χ^2^ = 0.03, *p* = 0.92), and music exposure or experiences. See **Table 1** for the means and standard deviations of the TCT-DP, arousal, and valence scores for the three groups.

**Table 1:** Means and standard deviations of the TCT-DP, arousal, and valence scores in the pre- and post-test conditions for the three groups.

	Pre-test		Post-test	
	*Mean*	*SD*	*Mean*	*SD*
*Positive music group* (*n* = 198)				
TCT-DP	18.9	9.89	22.3	11.7
Arousal	4.91	1.36	5.82	1.81
Valence	4.83	1.36	6.07	1.98
*Negative music group* (*n* = 195)				
TCT-DP	18.6	3.22	21.9	8.83
Arousal	4.86	1.50	6.00	1.77
Valence	4.88	1.42	3.68	1.23
*Control group* (*n* = 191)				
TCT-DP	18.5	10.5	19.0	8.84
Arousal	5.01	1.34	4.85	0.76
Valence	5.03	1.42	4.86	1.20


In the subsequent week, the two experimental groups were exposed to a stimulus condition (or control) for 10 min and then tested immediately afterward with the Affect Grid and the TCT-DP Form B. The stimulus conditions consisted of listening to Mozart (i.e., positive music group) or Albinoni (negative music group). The control group was instructed to sit in silence for 10 min.

## Results

### Manipulation Checks

**FIGURE 2:**
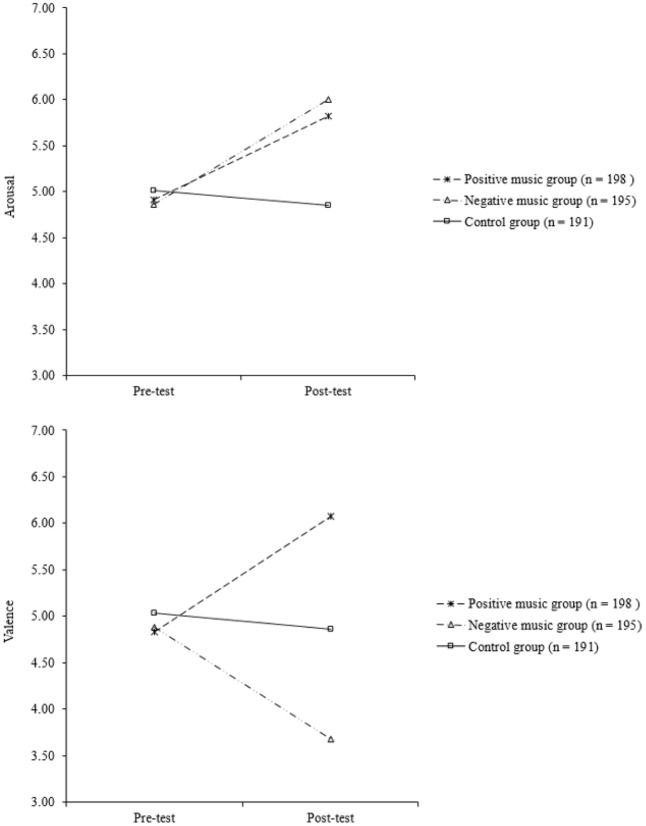
Changes in the arousal and valence scores between the pre- and post-test conditions for the three groups.

To illustrate whether the emotion-induction procedure was successful, a three groups by two time points repeated-measures analysis of variance (ANOVA) was conducted to examine whether the arousal and the valence scores changed significantly between the pre- and post-test conditions in the three groups. See **Figure 2** for the changing patterns of the arousal and valence scores between the pre- and post-test conditions in the three groups. The results of the repeated-measures ANOVA illustrated a significant group × time interaction effect for both arousal (Wilks’ Lambda = 0.89, *F*[2,581] = 34.3, *p* = 0.000, $\eta_{p}^{2}$ = 0.11) and valence (Wilks’ Lambda = 0.76, *F*[2,581] = 89.8, *p* = 0.000, $\eta_{p}^{2}$ = 0.24), suggesting that the changes in arousal and valence scores across the two time points were different among the three groups. Subsequent repeated-measures ANOVAs were thus performed separately for the three groups in both arousal and valence. An adjusted *p*-value of 0.017 (i.e., 0.05/3) was used to determine the significance level of the statistical tests.

The results of the repeated-measures ANOVA suggest that the emotion-induction procedure was successful in inducing emotional reactions. As expected, there was a statistically significant increase in the arousal scores between the pre- and post-test conditions in both the positive (Wilks’ Lambda = 0.74, *F*[1,197] = 67.7, *p* = 0.000, $\eta_{p}^{2}$ = 0.26) and the negative music groups (Wilks’ Lambda = 0.74, *F*[1,194] = 67.2, *p* = 0.000, $\eta_{p}^{2}$ = 0.26). No such significant change was observed in the control group (Wilks’ Lambda = 0.99, *F*[1,190] = 2.58, *p* = 0.11, $\eta_{p}^{2}$ = 0.01).

The emotion-induction procedure was also effective in inducing the corresponding valence of emotional reactions. The results of the repeated-measures ANOVA illustrated that the positive music group showed a statistically significant increase in the valence score (Wilks’ Lambda = 0.73, *F*[1,197] = 72.7, *p* = 0.000, $\eta_{p}^{2}$ = 0.27) between the pre- and post-test conditions. However, in contrast, the negative music group exhibited a statistically significant decrease in the valence score (Wilks’ Lambda = 0.70, *F*[1,194] = 81.9, *p* = 0.000, $\eta_{p}^{2}$ = 0.30). In the control group, again, no significant change was observed (Wilks’ Lambda = 0.99, *F*[1,190] = 2.31, *p* = 0.13, $\eta_{p}^{2}$ = 0.01).

### Testing the Effect of Music Listening on Creative Thinking

**FIGURE 3:**
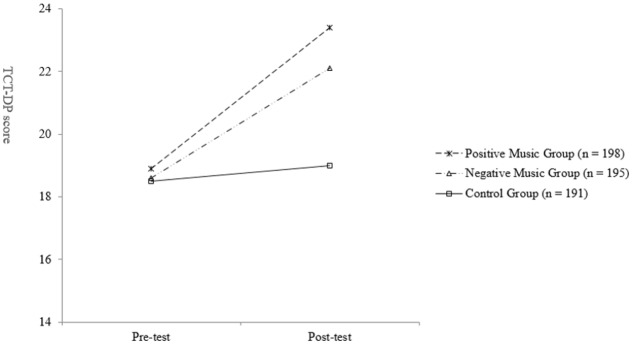
Changes in the TCT-DP scores between the pre- and post-test conditions for the three groups.

To test Hypothesis 1, which states that music listening enhances creative thinking, a repeated-measures ANOVA was conducted for the three groups (positive music group vs. negative music group vs. control group) with two time points (pre- vs. post-test). The changes in the TCT-DP scores between the pre- and post-test conditions among the three groups are presented in **Figure 3**. The results of the significant group × time interaction effect (Wilks’ Lambda = 0.99, *F*[2,581] = 4.17, *p* = 0.016, $\eta_{p}^{2}$ = 0.01) suggest that the change in TCT-DP across the two time points was different among the three groups. Hence, subsequent repeated-measures ANOVAs were performed for the three groups separately, and an adjusted *p*-value of 0.017 (i.e., 0.05/3) was used to determine the significance level of the statistical tests.

Lending support to Hypothesis 1, the results of the repeated-measures ANOVAs revealed a significant increase in the mean TCT-DP scores from the pre- to post-test time points for both the positive (Wilks’ Lambda = 0.94, *F*[1,197] = 13.2, *p* = 0.000, $\eta_{p}^{2}$ = 0.06) and negative music groups (Wilks’ Lambda = 0.86, *F*[1,194] = 30.4, *p* = 0.000, $\eta_{p}^{2}$ = 0.14). For the control group, however, no significant change was observed in the mean TCT-DP score over time (Wilks’ Lambda = 1.00, *F*[1,190] = 0.48, *p* = 0.49, $\eta_{p}^{2}$ = 0.00).

To further examine whether the three groups significantly differed in their performance on the post-test of the TCT-DP, a univariate ANOVA was conducted. The results suggest that the three groups demonstrated a significantly different performance in the TCT-DP on the post-test (*F*[2,581] = 6.41, *p* = 0.002, $\eta_{p}^{2}$ = 0.02). Further, the results of *post hoc* pairwise comparisons with the Bonferroni procedure, which was used to adjust for multiple comparisons, suggested that both the positive (*t* = 3.30, *p* = 0.003) and negative (*t* = 2.95, *p* = 0.011) music groups obtained a significantly higher TCT-DP score than the control group on the post-test. There was no statistically significant difference between the positive and negative music groups on their TCT-DP scores in the post-test (*t* = 0.35, *p* = 1.00), suggesting that listening to both positively and negatively inducing music has no statistically significant effect on performance on the TCT-DP.

### Testing the Mediation Effect of Emotional Reactions on the Music-Creativity Relation

**FIGURE 4:**
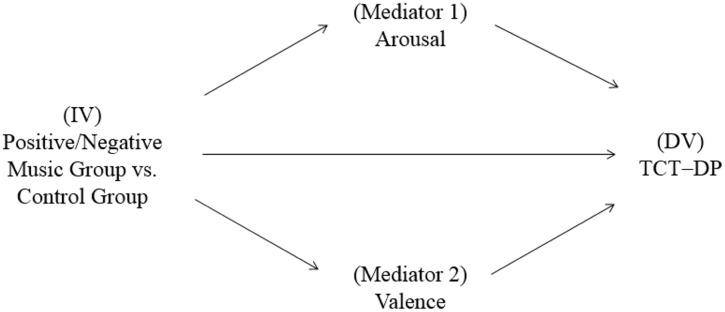
The proposed mediation model.

To test Hypothesis 2, which postulates that emotional reactions (i.e., arousal and valence) mediate the effect of music listening on creative thinking, Preacher and Hayes’ (2008) test of mediation effects was used. The proposed mediation model is presented in **Figure 4**, which illustrates that arousal and valence served as the mediators (Mediators), the condition of music listening vs. sitting silence served as the independent variable (IV), and the TCT-DP score on the post-test served as the dependent variable (DV). Prior to the examination of the mediation effect, multicollinearity was assessed by measuring the formal detection tolerance and the variance inflation factor (VIF). The results of multiple regressions showed that multicollinearity was not a problem, with the tolerance values of all predictor variables greater than 0.80 and all VIF values smaller than 1.10 (Mertler and Reinhart, 2017). Subsequently, the mediation analysis was conducted by using Hayes’ PROCESS procedure for SPSS (Model 4; Preacher and Hayes, 2008). A total of 5,000 bootstrap samples were used to create 95% bias-corrected and accelerated bootstrap confidence intervals (CIs) of the indirect effect. The results are summarized below.

#### Effect of Listening to Positively Arousing Music

**FIGURE 5:**
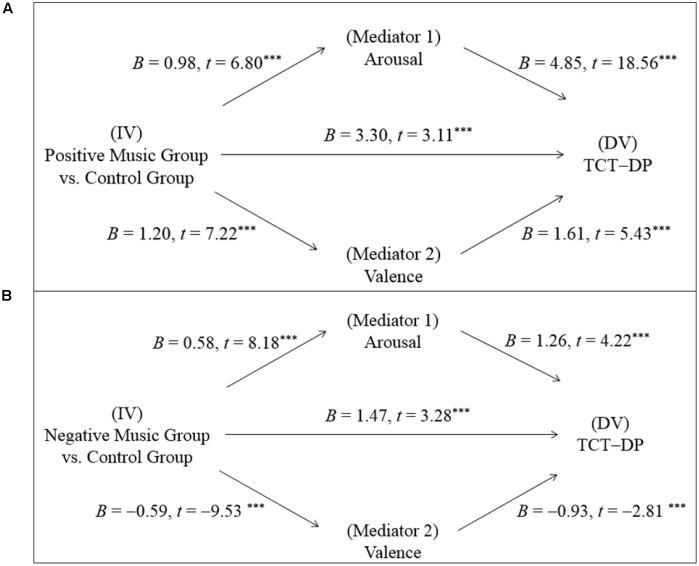
Path of music listening on creative thinking mediated by arousal and valence in the conditions of **(A)** listening to positively arousing music and **(B)** listening to negatively arousing music. ^∗∗∗^*p* < 0.001.

**Figure 5A** summarizes the results regarding the relationships among the three variables (i.e., music listening, emotional reactions, creative thinking) in the condition of listening to positive music. The results illustrated that listening to positive music (IV) had a significant effect on both the DV (TCT-DP: *B* = 3.30, *SE* = 1.06, *t* = 3.11, *p* = 0.002) and the two hypothesized mediators (Arousal: *B* = 0.98, *SE* = 0.14, *t* = 6.80, *p* = 0.000; Valence: *B* = 1.20, *SE* = 0.17, *t* = 7.22, *p* = 0.000). Moreover, the two hypothesized mediators demonstrated significant effects on the DV (TCT-DP; Arousal: *B* = 4.85, *SE* = 0.26, *t* = 18.56, *p* = 0.000; Valence: *B* = 1.61, *SE* = 0.30, *t* = 5.43, *p* = 0.000).

With respect to the mediation effect, the results of the mediation analysis using Hayes’ PROCESS procedure for SPSS suggested that arousal was a significant mediator, with the mean of the indirect effect across all bootstrap samples estimated at 3.01 (*SE* = 0.57) and a resulting confidence interval that did not include 0 (CI [2.00, 4.24]). However, the results of the mediation analysis suggest that valence was not a significant mediator, with the mean of the indirect effect across all bootstrap samples estimated at 0.51 (*SE* = 0.39) and a resulting confidence interval that did include 0 (CI [-0.19, 1.33]).

#### Effect of Listening to Negatively Arousing Music

In the condition of listening to negative music, the same statistical procedure was used to determine whether arousal and valence mediate the music-creativity relationship (see **Figure 5B** for a summary of the results). The results illustrated that listening to negative music (IV) had a significant effect on both the DV (TCT-DP: *B* = 1.47, *SE* = 0.45, *t* = 3.28, *p* = 0.001) and the two hypothesized mediators (Arousal: *B* = 0.58, *SE* = 0.07, *t* = 8.18, *p* = 0.000; Valence: *B* = -0.59, *SE* = 0.06, *t* = -9.53, *p* = 0.000). Moreover, the two hypothesized mediators demonstrated significant effects on the DV (TCT-DP; Arousal: *B* = 1.26, *SE* = 0.30, *t* = 4.22, *p* = 0.000; Valence: *B* = -0.93, *SE* = 0.33, *t* = -2.81, *p* = 0.005).

The results regarding the mediation effect were similar to those reported for the condition of listening to positive music. Again, arousal was shown to be a significant mediator, with the mean of the indirect effect across all bootstrap samples estimated at 3.55 (*SE* = 0.57) and a resulting confidence interval that did not include 0 (CI [2.57, 4.80]). However, valence was not a significant mediator, with the mean of the indirect effect across all bootstrap samples estimated at -0.50 (*SE* = 0.36) and a resulting confidence interval that did include 0 (CI [-1.22, 0.21]).

## Discussion

The present study examined the effect of music listening on creative thinking through the lens of the arousal-and-mood hypothesis, which postulates that emotional reactions mediate the effect of music listening on the subsequent performance of cognitive functioning (Husain et al., 2002; Schellenberg, 2005; Schellenberg et al., 2007). Lending supports to Hypothesis 1, which states that music listening enhances creative thinking, the results of the repeated-measures ANOVA suggest that listening to both positively and negatively arousing music enhances creative thinking. Lending partial support to Hypothesis 2, which states that emotional reactions (i.e., arousal and valence) mediate the effect of music listening on creative thinking, the results of the mediation analyses using Hayes’ PROCESS procedure for SPSS suggest that only arousal, and not valence, mediates the music-creativity relationship in both conditions, i.e., listening to positively or negatively arousing music. Overall, the findings of this study lend partial support to the arousal-and-mood hypothesis in the creativity domain. The findings enrich the discourse of the arousal-and-mood hypothesis and may shed light on the understanding of the effect of music listening on creative thinking. Some important contributions and interesting findings of the present study are highlighted below.

### The Mediating Roles of Emotions in the Music-Creativity Relationship Are Directly Assessed

One significant contribution of the present study is its direct measure of the mediating roles of emotions (i.e., arousal and valence) in the relationship between music and creativity. Whereas many studies in the literature have aimed to provide empirical support to the arousal-and-mood hypothesis (e.g., Thompson et al., 2001; Husain et al., 2002; Schellenberg et al., 2007), such studies have presented results that showed merely parallel relationships between (1) music exposure and task performance and (2) music exposure and emotional reactions. Specifically, these previous studies demonstrated that participants who scored higher on arousal and positive valence of emotional reactions after listening to music also demonstrated a parallel improvement in subsequent task performances; however, no effect of the music was shown for participants who scored low on arousal and valence of emotional reactions. While showing the parallel relationships among the three variables of music exposure, emotional reaction, and task performance might suggest indirect empirical support for the arousal-and-mood hypothesis, the present study presented direct empirical evidence with respect to the hypothesis by (1) employing a standardized measure of emotional reactions (i.e., the Affect Grid) to assess changes in arousal and valence in response to the music listening experience and (2) applying Hayes’ statistical procedure (Preacher and Hayes, 2008) to determine the mediation effect of arousal and valence on the relationship between music and creativity.

The contribution of direct evidence for the mediation effects of emotional reactions on the music-creativity relationship may also enrich the literature with respect to the relations among music, emotions, and creativity. There are three separate lines of research findings in the existing literature regarding the relations among music, emotions, and creativity, which suggest (1) a positive role of music exposure in creativity; (2) a positive effect of music exposure on emotion inductions, and (3) a positive effect of emotion inductions on creativity. The finding of direct evidence for the mediation effect of emotional reactions on the music-creativity relationship supports a meaningful integration of these three separate lines of research. The finding also enriches the understanding of the underlying psychological mechanisms that explain the effect of music exposure on creativity.

### Both Positive and Negative Valence Enhance Creative Thinking

The second contribution of the study is its enrichment of the research regarding the arousal-and-mood hypothesis by including both positively and negatively arousing music. While the arousal-and-mood hypothesis focuses merely on positive emotions and postulates that any optimally arousing stimulus that induces positive moods should enhance performance on a variety of cognitive tasks, the findings of our study show that both positive and negative emotions can enhance creative thinking, as long as the music experience can successfully arouse an individual’s emotion. These findings are important because they suggest that the generalizability of the arousal-and-mood hypothesis is not limited to positively aroused emotions; rather, it can be extended to negatively aroused emotions. These findings are also important to the mood-creativity literature, in which inconsistent findings are often documented with respect to the effect of negative emotions on creativity (Kaufmann and Vosburg, 1997; George and Zhou, 2002; Gasper, 2003; Shalley et al., 2004; Jovanovic et al., 2016), although more consistent findings are reported regarding the facilitative role of positive emotions in creativity (e.g., Isen et al., 1987; Fredrickson, 1998; Ashby et al., 1999; Lyubomirsky et al., 2005; Isen, 2008). The findings of the current study provide empirical support to the theories that argue that both positive and negative emotions can function on creativity but through different routes or processes (Schwarz, 1990; Baas et al., 2008, 2011; De Dreu et al., 2008; Davis, 2009).

### Arousal, but not Valence, Mediates the Music-Creativity Link

The third contribution of this study concerns the finding that only arousal, and not valence, was a significant mediator of the music-creativity link. This finding is not entirely consistent with the prediction of the arousal-and-mood hypothesis, which posits that both arousal and valence play a parallel and equally important role in mediating the relationship between music exposure and cognitive functioning. However, our findings seem to accord with De Dreu et al.’s (2008) dual pathway to creativity model, which suggests different roles of arousal (or activation) and valence (hedonic tone) in the mediation paths in the mood-creativity link. In this model, arousal is regarded as the necessary precondition or the critical entry point of the mediation path. Valence (positive or negative), at the second step, determines the subsequent paths through which creativity is achieved. In De Dreu et al.’s (2008) words, “activation is a necessary precondition for creativity to come about and that hedonic tone determines the route—the flexibility route or the perseverance route—through which creative fluency and originality is achieved” (p. 740). They further elaborate that “the level of activation associated with a particular mood state serves as the critical entry point, with higher activation leading to greater fluency and originally. However, which pathway is used depends on a mood state’s hedonic tone, with positive tone facilitating the cognitive flexibility route and negative tone facilitating the cognitive perseverance route” (De Dreu et al., 2008, p. 742).

The critical role of arousal has also been supported by research demonstrating that emotional states high in arousal and valence (i.e., happy) are associated with more creativity via increasing cognitive flexibility (i.e., broad attention, accessing multiple cognitive categories), whereas emotional states low in arousal but high in valence (i.e., relaxed) are not related to greater creativity. The research findings further illustrate that although emotional states high in arousal but low in valence (i.e., anger, fear) decrease flexibility, such emotional states could increase creativity by stimulating perseverance. However, emotional states low in both arousal and valence (e.g., sadness) do not lead to creativity (Baas et al., 2008; Nijstad et al., 2010). These research findings underscore the important role of arousal, regardless of valence, in creativity.

With respect to the role of arousal, the arousal-and-mood hypothesis highlights that a moderate level of arousal is critical (Husain et al., 2002; Schellenberg, 2005; Schellenberg et al., 2007). This theoretical notion is supported by the findings of the present study, which revealed that the arousal level on the post-test was 5.82 and 6.00 in the conditions of listening to positive and negative music, respectively. The scores of 5.82 and 6.00 can be regarded as moderate on a scale ranging from 1 (lowest) to 9 (highest) in the Affect Grid. This finding aligns with the view that that neither low levels of arousal nor extremely high levels of arousal contribute to effective cognitive functioning (Yerkes and Dodson, 1908; Staw et al., 1981; Carnevale and Probst, 1998; Berridge and Waterhouse, 2003). It is suggested that low levels of arousal may lead to inactivity, avoidance, and neglect of information, which lower cognitive performance. In contrast, extremely high levels of arousal may reduce the capacity to perceive, process, and evaluate information, which also hinder effective cognitive functioning. Only at moderate levels of arousal may individuals demonstrate an optimum level of performance by showing a high level of motivation to seek and integrate information and to consider multiple alternatives (Baas et al., 2008; De Dreu et al., 2008).

### The Arousal-and-Mood Hypothesis Is Partially Supported in the Creativity Domain

Overall, the findings of the present study can be regarded as lending partial support to the arousal-and-mood hypothesis. Past studies regarding the arousal-and-mood hypothesis have been mainly conducted in the intelligence domain (predominantly in the spatial intelligence domain). The current study extends this line of study and suggests that the hypothesis can be generalized to the creativity domain. If listening to music benefits only a restricted set of tasks, as was shown in past studies (i.e., spatial reasoning), then the effect of music would be limited as a tool designed to improve spatial ability in educational and practical settings. However, if listening to music benefits creative thinking, such findings may shed light on creativity education. Most existing creativity-training programs are based on thinking techniques (e.g., six thinking hats, mind maps, creative problem-solving skills); the idea of using music to improve creativity seems to be unusual in the field. However, music exposure has advantages over other thinking techniques in nurturing creativity. For example, listening to music is an activity that can be enjoyed by individuals of all ages and stages of development. Moreover, music listening requires little verbal skill, so it can be used as a creativity-training technique for individuals who have language difficulties.

### Limitations and Future Research

We note several limitations. First, although the arousal-and-mood hypothesis generalizes that any pleasant or enjoyable stimulus, either musical or non-musical, that arouses positive mood can enhance a wide range of cognitive functions, we focused merely on musical stimuli. Specifically, our stimuli were only classical music. Future empirical scrutiny on the hypothesis should be generalized to other types of musical stimuli and also to non-musical stimuli. Second, creativity was assessed with only a single measure of creativity (i.e., the TCT-DP). Although the TCT-DP has been suggested to be a reliable and valid test of creative thinking, it was designed to tap into an individual’s creative potential. It remains an open question as to whether the findings of this study can be replicated if other creativity tests are used.

Third, a clear test of the prediction that any arousing music piece would enhance creativity may require a condition in which participants listen to music that lowers arousal levels (e.g., relaxing music). Future research should address this issue by including relaxing music. Fourth, participants in this study first listened to music and subsequently performed a creativity task. Future research should explore whether similar or different results will be found if participants work on a creativity task while simultaneously listening to music. Future research should also explore whether similar or different results will be found in other cognitive domains. Fifth, it is notable that the effect sizes found in this study were only small to medium. This may be because the duration of music exposure was brief (i.e., 10 min). It is worthwhile to further investigate whether a longer period of music exposure would increase the effect sizes.

## Conclusion

Despite the above-mentioned limitations, the present study enriches the current research on the arousal-and-mood hypothesis, which is helpful in explaining many phenomena regarding the effects of music, such as for whom and under what circumstances listening to music enhances cognitive functioning and what type of music is effective. Demonstrating that the effect of music exposure is the result of an individual’s emotional reaction to musical stimuli helps to resolve a theoretically intriguing issue in the field, and it explains why some studies support the idea that listening to a piece of music composed by Mozart or other composers can enhance cognitive functioning when others cannot. Such findings imply that the pedagogical benefits of music depend more on the interactions between individual factors and the type or style of music. Given that not all individuals benefit equally from all types of music, individuals might benefit from any piece of music that provides optimal arousal and evokes either a positive or a negative hedonic tone. Simplistic solutions such as the Mozart effect can give false impressions and unrealistic expectations. Highlighting the nature and the quality of music exposure and searching for the optimal music that arouses an individual’s appropriate emotional reactions would be more important for the facilitation of cognitive functioning.

## Ethics Statement

This study was carried out in accordance with the recommendations of the Operational Guidelines and Procedures of the Human Research Ethics Committee of the Hong Kong Institute of Education with written informed consent from all subjects. All subjects gave written informed consent in accordance with the Declaration of Helsinki. The protocol was approved by the Human Research Ethics Committee of the Hong Kong Institute of Education.

## Author Contributions

W-JH contributed to the conception and design of the work as well as the acquisition, analysis, and interpretation of the data. She also drafted, revised, and approved the final version of the manuscript. W-CW contributed to the conception and design of the work as well as the revisions and approvals of the final version of the manuscript. AH contributed to the conception of the work as well as the revisions and approvals of the final version of the manuscript. All authors agree to be accountable for the content of the work.

## Conflict of Interest Statement

The authors declare that the research was conducted in the absence of any commercial or financial relationships that could be construed as a potential conflict of interest.
